# Quantitative evaluation of motor function before and after engraftment of dopaminergic neurons in a rat model of Parkinson's disease

**DOI:** 10.1186/1423-0127-17-9

**Published:** 2010-02-13

**Authors:** Chieh-Sen Chuang, Hong-Lin Su, Fu-Chou Cheng, Shan-hui Hsu, Chi-Fen Chuang, Chin-San Liu

**Affiliations:** 1 Department of Neurology, Changhua Christian Hospital, Changhua City 500, Taiwan; 2 Department of Life Sciences, National Chung-Hsing University, Taichung City, 40227, Taiwan; 3 Department of Medical Research, Taichung Veterans General Hospital, Taichung City 407, Taiwan; 4 Institute of Polymer Science and Engineering, National Taiwan University, Taipei City 106, Taiwan; 5 Vascular and Genomic Centre, Changhua Christian Hospital, Changhua City 500, Taiwan; 6 Graduate Institute of Integrative Chinese and Western Medicine, China Medical University, Taichung City 404, Taiwan

## Abstract

Although gait change is considered a useful indicator of severity in animal models of Parkinson's disease, systematic and extensive gait analysis in animal models of neurological deficits is not well established. The CatWalk-assisted automated gait analysis system provides a comprehensive way to assess a number of dynamic and static gait parameters simultaneously. In this study, we used the Catwalk system to investigate changes in gait parameters in adult rats with unilateral 6-OHDA-induced lesions and the rescue effect of dopaminergic neuron transplantation on gait function. Four weeks after 6-OHDA injection, the intensity and maximal area of contact were significantly decreased in the affected paws and the swing speed significantly decreased in all four paws. The relative distance between the hind paws also increased, suggesting that animals with unilateral 6-OHDA-induced lesions required all four paws to compensate for loss of balance function. At 8 weeks post-transplantation, engrafted dopaminergic neurons expressed tyrosine hydroxylase. In addition, the intensity, contact area, and swing speed of the four limbs increased and the distance between the hind paws decreased. Partial recovery of methamphetamine-induced rotational response was also noted.

## Introduction

Parkinson's disease (PD) is the second most common neurodegenerative disorder after Alzheimer's disease. The worldwide prevalence is estimated to be 200 per 100,000 population [1]. Degeneration of dopamine neurons in the substantia nigra (SN) and the consequent deficit of dopamine release in the striatum and other target areas appear to be responsible for the characteristic manifestations of PD. Common parkinsonian symptoms are rest tremor, bradykinesia, rigidity, and loss of postural reflexes [2]. Gait disturbances are one of the most common motor problems in Parkinson's disease. Patients with PD often present with a stooped posture and shuffling gait, decreased stride length and overall velocity, increased double-limb support, reduced foot clearance during swing phase, and increased cadence leading to the potential for falls [3-5]. Progressive gait disturbance combined with posture instability finally deprives patients of locomotor ability and activities of daily living [6]. In typical cases, however, the onset of symptoms is asymmetrical, with tremor and rigidity affecting limbs on one side of the body first. Although the limbs on the contralateral side of the body will eventually be affected, it can often be several years before the symptoms manifest.

Although systematic gait analyses have been widely used in the clinical setting as important indices to evaluate the severity of PD, the extent of gait changes after unilateral 6-OHDA-induced lesions in rats remains to be explored. One of the methods of analyzing gait and ambulation in animal models of PD includes the treadmill locomotion test to evaluate walking velocity, swing and stance time [7]. Other methods include the cylinder test to assess forelimb-use asymmetry and the forelimb akinesia test to measure movement initiation in animals with 6-OHDA-induced lesions [8,9]. These tests, however, measure only dynamic or static changes in gait. Therefore, multiple methods are necessary to evaluate dynamic and static gait parameters simultaneously. A computer-assisted gait analysis, called the CatWalk method, provides an automated way to assess gait function with the benefit of measuring a large number of both dynamic and static gait parameters simultaneously [10]. The CatWalk method can also detect the spatial and temporal aspects of inter-limb coordination that are particularly valuable for rodent studies. This method has been used in a variety of quadrupedal studies for assaying impaired gait function after spinal cord injury [11], pyramidotomy [12], stroke [13] and drug-induced neuropathy [14].

The pathophysiology of PD is characterized by the degeneration of dopamine neurons and a reduction in striatal dopamine release. PD is a slowly progressive disorder without a known cure. The most effective therapy is the administration of the dopamine precursor levodopa or dopamine agonists. As the disease progresses, however, the beneficial effect of these drugs may diminish and become less consistent. Dopamine replacement by grafting exogenous cells may provide better long-term results. Stem cells are viewed as a possible source of neurons for cell-based therapies of neurodegenerative disorders, such as Parkinson's disease and Alzheimer's disease [15]. Neural stem cells that have been transplanted into 6-OHDA-lesioned rats have been shown to differentiate into neuronal cell types that express markers of dopaminergic neurons such as tyrosine hydroxylase (TH) and aromatic amino acid decarboxylase (AADC) [16]. In the present study, we used the CatWalk-assisted automated gait analysis system to evaluate gait changes before and after transplant of dopamine neurons derived from embryonic stem cells (ES cells) in a unilateral 6-OHDA rat model of PD.

## Materials and methods

### Animals and housing

Adult female Sprague-Dawley rats (8 weeks old, weighing 250-300 g) were housed in standard cages under conditions of controlled temperature (23 ± 3°C) and in a regular light-dark cycle. Animals were given free access to standard rat chow and water. Rats (n = 24) were randomly assigned to receive 6-OHDA injection (n = 14) or sham surgery (n = 10). All experimental procedures were approved by the Animal Experiments and Ethics Committee of the Changhua Christian hospital, Taiwan. The experiments were designed to minimize the number of animals used and their suffering.

### Rat model of PD

Female Sprague-Dawley rats weighing 250-300 g were placed in a stereotactic apparatus. Burr holes were drilled in the right side of the skull and 30 μg 6-OHDA or 0.9% saline (sham) was injected into the ascending mesostriatal pathway (4.4 mm posterior to the bregma, 1.2 mm lateral to the midline, 7.8 mm below the dura) near the medial forebrain bundle to remove dopaminergic innervation to the striatum [17,18].

### Rotational behavior test

Motor imbalance in 6-OHDA-lesioned animals was assayed by methamphetamine (3 mg/kg, ip)-induced rotation, as described by Ollson [18]. The rats were placed on a multi-channel motor meter connected to a computer to record clockwise or counter clockwise rotation. Methamphetamine-induced rotation was evaluated at 4 weeks after the 6-OHDA injection. Rats that rotated over 360 turns in 60 minutes were chosen and randomly assigned for implantation of ES-derived dopamine neurons (n = 5) or injection of vehicle for control (n = 5). The methamphetamine-induced rotation was evaluated again at four and eight weeks after transplantation or injection of vehicle.

### Stem cell culture

Sox1-GFP knock-in ES cells (46C), a kind gift from Dr. Austin Smith (University of Cambridge, UK) [19], were maintained in 0.1% gelatin-coated culture dishes in high glucose DMEM supplemented with 1% fetal bovine serum, 10% Knock-out serum replacement, 2 mM glutamine, 0.1 mM nonessential amino acids, 1 mM pyruvate, 0.1 mM 2-mercaptoethanol (Sigma), and 1000 U/ml Leukemia inhibitory factor (LIF, Chemicon). ES cells were patterned to become midbrain dopaminergic neurons by adding sonic hedgehog (200 ng/ml, R&D) and FGF8b (40 ng/ml, R&D) to the differentiation medium on day 5 in the presence of serum-free embryoid bodies (SFEB). On differentiation day 10, ES-derived dopaminergic neurons were used for transplantation.

### Stem cell transplantation

At four weeks after 6-OHDA lesioning, animals that rotated over 360 turns in a 60-minute period during the rotational test were chosen for implantation of ES-derived dopamine neurons (n = 5) or vehicle injection (n = 5). Rats were anesthetized with chloral hydrate (400 mg/kg, i.p.) and then placed in a Kopf stereotaxic frame (Kopf Instruments, Tujunga, CA) for injection. Animals received 0.5 μl/min of ES-derived doaminergic neurons (about 25 × 10^4 ^ES cells) or saline administered into the right striatum (from the bregma: anteroposterior (AP), ± 0.0 mm; lateriomedial (LM) +3.0 mm; dorsoventral (DV) -5.0 mm and AP ± 0.0 mm, LM +3.0 mm, DV -4.5 mm) using a 26-gauge, 10-μl Hamilton syringe. There was a 5-min waiting period before the needle was removed. Animals in the control group received a total of 5 μl ES (25 × 10^4 ^ES cells) and animals in the control group received a total of 5 μl saline. All animals were given cyclosporine A (10.0 mg/kg i.p., Novartis Pharma) daily for immunosuppressive.

### Computer-assisted method for gait analysis

Rats were subjected to gait assessment with the CatWalk-automated gait analysis system (Noldus Information Technology, Wageningen, The Netherlands) before 6-OHDA-lesioning or sham operation. The rats that had received 6-OHDA were evaluated again 4 weeks after 6-OHDA injection and at 8 weeks after transplantation or injection of vehicle. The apparatus comprises a long glass plate with a fluorescent light beamed into the glass walkway floor from one side. In a dim environment, the light is reflected downward and the footprints of the rat as it walks along the walkway are recorded by a camera mounted under the glass.

### Gait parameters

The gait parameters examined with the CatWalk software in this study are described below (LF, left forepaw; LH, left hindpaw; RF, right forepaw; RH, right hindpaw).

#### Base of support (BOS)

This parameter is an indication of double-limb support. It is derived by measuring the distance (mm) between the mass-midpoints of the two forelimb prints or the two hindlimb prints at maximum contact.

#### Intensity

This parameter is an indication of paw pressure. It is an indirect measure of the mean pressure applied at the moment of floor contact (arbitrary unit, a.u.).

#### Swing speed

This parameter refers to the velocity of the moving limb during the swing phase (m/s). It is computed from stride length and swing duration.

#### Max area

This parameter is a measure of the surface area of maximal contact of the paw with the ground (pixel^2^).

#### Phase dispersion

**Figure 1:**
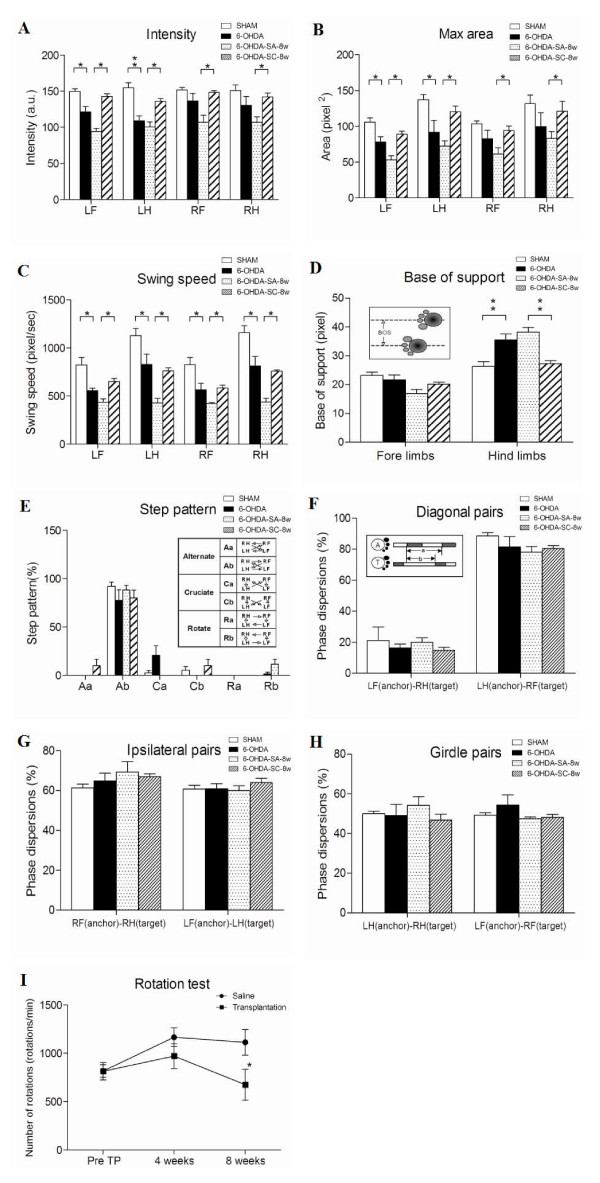
**Effect of dopaminergic depletion and embryo stem cell derived dopaminergic neuron implantation**. Gait parameters were assessed at 4 weeks after injection of 6-OHDA (6-OHDA) or saline (SHAM) at 8 weeks after transplantation of ES-derived dopaminergic neurons (6-OHDA-SC-8w) or injection of vehicle (6-OHDA-SA-8w) as the control. The intensity and max area (A and B) were reduced in the left paws and the swing speed (C) of all paws decreased after 6-OHDA injection. After implantation of dopaminergic neurons, the intensity, max area, and swing speed of all paws improved. The BOS between the hind paws increased after injection of 6-OHDA and partially recovered after dopaminergic neuron implantation (D). 6-OHDA-lesioning and dopaminergic neuron implantation did not lead to a significant change in step patterns and inter-limb phase dispersions (E, F, G and H). Rotational behavior in response to methamphetamine was tested at pre-transplantation (pre-TP) and at 4 and 8 weeks post-grafting. A significant decrease in absolute numbers of drug-induced turning was seen in the transplantation group compared with control animals at eight weeks (I). *P < 0.05; **P < 0.005.

This parameter is a measure of the temporal relationship between placement of two paws within a step cycle. Phase dispersion depends on the initial contact of one paw (target paw) to the stride cycle of another paw (anchor paw) and is expressed as a percentage (in Figure 1F, Phase dispersion is calculated by b/a * 100%). Phase dispersion can be calculated between the paws of the same girdle, between paws on the same side, or between diagonal paws. It is used as a measure of inter-paw coordination.

#### Step pattern

There are a total of six possible step sequence patterns that a rat can use as it places its four paws one after another (Figure 1E). These patterns can be categorized into 3 groups: alternate (Aa: RF-RH-LF-LH; Ab: LF-RH-RF-LH; cruciate (Ca: RF-LF-RH-LH; Cb: LF-RF-LH-RH); and rotary (Ra: RF-LF-LH-RH; Rb: LF-RF-RH-LH).

#### Regularity index (RI)

The regularity index expresses the number of normal step sequence patterns relative to the total number of paw placements. It is a percent index and is used as a measure of the degree of inter-limb coordination during the gait cycle.

### Immunocytochemistry and Histology

At four weeks after injection of 6-OHDA or saline, one animal in the experimental group and one animal in the sham group were killed by an i.p. overdose of chloral hydrate (800 mg/kg i.p.) perfused intracardially with 300 ml saline followed by 300 ml of paraformaldehyde (4% in PBS). The brain was removed from each rat, post-fixed in 4% paraformaldehyde for 4 hours, and cryoprotected in 30% w/v sucrose in PBS for 20 hours. The brains were then frozen and stored at -80°C. The tissues were embedded in Tissue-Tek OCT medium and cryosectioned at 30-μm thickness. The tissues were then incubated overnight at 4°C with monoclonal antibodies against tyrosine hydroxylase (TH, 1:200; Mouse anti-tyrosine hydroxylase; chemicon) to evaluate the level of dopaminegic depletion in the substantia nigra. At 8 weeks after implantation of ES-derived dopamine neurons or injection of vehicle (control), animals in the experimental group and in the control group were killed with an overdose of chloral hydrate (800 mg/kg i.p.). Brains were removed and then incubated overnight with primary antibodies against M2 mouse-specific antibody (M2, 1:200; Rat anti-mouse cell surface, Developmental Studies Hybridoma Bank), DAPI (4',6-diamidino-2-phenylindole dihydrochloride (Merck) 1 ug/ml in ddH_2_O), and anti-TH for dopaminergic neurons. The immunostained sections were examined by light microscopy.

### Statistical analysis

All individual walkway crossings were analyzed using CatWalk software. Each locomotion parameter in each group is expressed as mean ± s.e.m for each condition. All statistical tests were performed with SPSS statistical software (version 15). Statistical significance was evaluated using one-way ANOVA and group differences were determined by the Mann-Whitney U test. A *P *value lower than 0.05 was considered to be statistically significant.

## Results

### Growth status among groups

The pre-surgical mean body weight of rats in the sham group was 270.2 ± 5.6 g and that of those in the 6-OHDA lesioning group was 272.0 ± 9.6 g (P < 0.001). At four weeks after lesioning, the mean body weight was 279.9 ± 11.3 g in the sham group and 281.4 ± 9.5 g in the lesioning group (P < 0.001). Before transplantation, the mean body weight of animals in the control group was 283.4 ± 8.9 g and that in the engrafting group was 278.8 ± 7.6 g (P < 0.01). At 8 weeks after transplantation, the mean body weight was 298.3 ± 15.4 g in the control group and 301.4 ± 21.5 g in the engrafting group (P < 0.01).

### Effects of 6-OHDA lesioning and of stem cell transplantation on gait parameters

#### Intensity

The signal intensity generated during placement of the paw is an estimate of paw pressure. Four weeks after injecting 6-OHDA or vehicle into the right ascending mesostriatal pathway, the pressure induced by each paw during floor contact, as measured by the intensity of the footprint, was lower in 6-OHDA-lesioned rats than in sham rats. However, a significant decrease in foot print intensity was only detected in the left paws (LF: 149.7 ± 3.9 vs 121.4 ± 7.4, P < 0.05; LH: 154.8 ± 7.1 vs 108.8 ± 6.9, P < 0.005). Eight weeks after transplantation of stem cells in the 6-OHDA-lesioned group, the signal intensity was significantly increased (Figure 1A), indicating that the paw pressure of all four limbs improved after engrafting ES-derived dopaminergic neurons.

#### Max area

The size of the print area at maximal contact of all four paws during floor contact was smaller in rats 4 weeks after 6-OHDA-lesioning than in sham rats; however, a significant decrease was detected only in the left paws (LF: 105.9 ± 5.8 vs 78.0 ± 7.4, P < 0.05; LH: 137.1 ± 7.3 vs 91.9 ± 16.5, P < 0.05). The size of the print area of all four paws was significantly greater in the experimental group at eight weeks after dopaminergic neuron transplantation than in the control group (Fig 1B.).

#### Swing speed

Swing speed refers to the velocity of a moving limb during the swing phase. We evaluated the effect of dopaminergic lesions on the velocity during the swing phase (pixel/sec). We found that the hind limbs moved faster than the fore limbs during the swing phase before administration of 6-OHDA. The velocity of the moving limb during the swing phase was lower after 6-OHDA injection. The swing speed of bilateral paws increased after dopaminergic neuron transplantation (Figure 1C.).

#### Base of support (BOS)

Base of support is the distance of the average width between either the front paws or the hind paws. We found that the distance between the two hind limbs was greater in 6-OHDA-lesioned rats than in sham rats (26.4 ± 1.6 vs 35.5 ± 2.1, P < 0.005). Our data showed that the BOS of the posterior paws was significantly reduced in rats that underwent neuron transplantation (38.2 ± 1.6 vs 27.3 ± 0.9, P < 0.005), indicating that the double-limb support of both hind paws improved in rats that received ES-derived dopaminergic neurons (Figure 1D).

#### Step pattern

There are six different regular step patterns described in rodents (Figure 1E). The most commonly observed pattern in rats in this study was the alternate pattern Ab (Ab: LF-RH-RF-LH), which accounted for 77.5% ± 11.1 of all regular step patterns in the 6-OHDA-lesioned rats and for 92.2% ± 4.11 in the sham rats. The cruciate gait pattern Ca (Ca: RF-LF-RH-LH) was noted in 20.8% of rats in the 6-OHDA group (Figure 1E). There were no significant changes in gait pattern after transplantation of dopaminergic neurons.

#### Regularity Index

The regularity index is the degree to which animals use normal step sequence patterns. It is expressed as the number of normal step sequence patterns relative to the total number of paw placements. This index is a measure of interlimb coordination. There was no significant difference in the regularity index between the sham rats (99.6 ± 0.4) and the rats that received 6-OHDA (95.4 ± 2.9) (P > 0.23).

#### Phase dispersion

The timed relationship between footfalls of two paws is a measure of inter-limb coordination. Phase dispersion reflects the time lag of initial contact of the target paw to the anchor paw. There were no differences in the phase dispersions for the diagonal pairs (RF_anchor_-LH_target _and LF_anchor_-RH_target_, Figure 1F), ipsilateral pairs (RF_anchor_-RH_target _and LF_anchor_-LH_target_, Figure 1G), or girdle pairs (LH_anchor_-RH_target _and LF_anchor_-RF_target_, Figure 1H) between rats that received saline and those that received 6-OHDA.

#### Rotational behaviour test

Rotational behavior is a useful indicator of unilateral dopaminergic lesions by 6-OHDA. The rotational response to methamphetamine was examined at 4 and 8 weeks after transplantation (experimental group) or infusion of vehicle (control group). The number of methamphetamine-induced rotations during a 60 min period for control animals and experimental animals is shown in Figure 1I. At 8 weeks after transplantation or infusion of vehicle, the number of rotations in animals that received ES-derived dopaminergic neurons was significantly lower than in control animals (P < 0.05), indicating the recovery of dopaminergic function.

### Sonic hedgehog with FGF8b enhanced the differentiaton of Sox1-GFP knock-in ES cells

**Figure 2:**
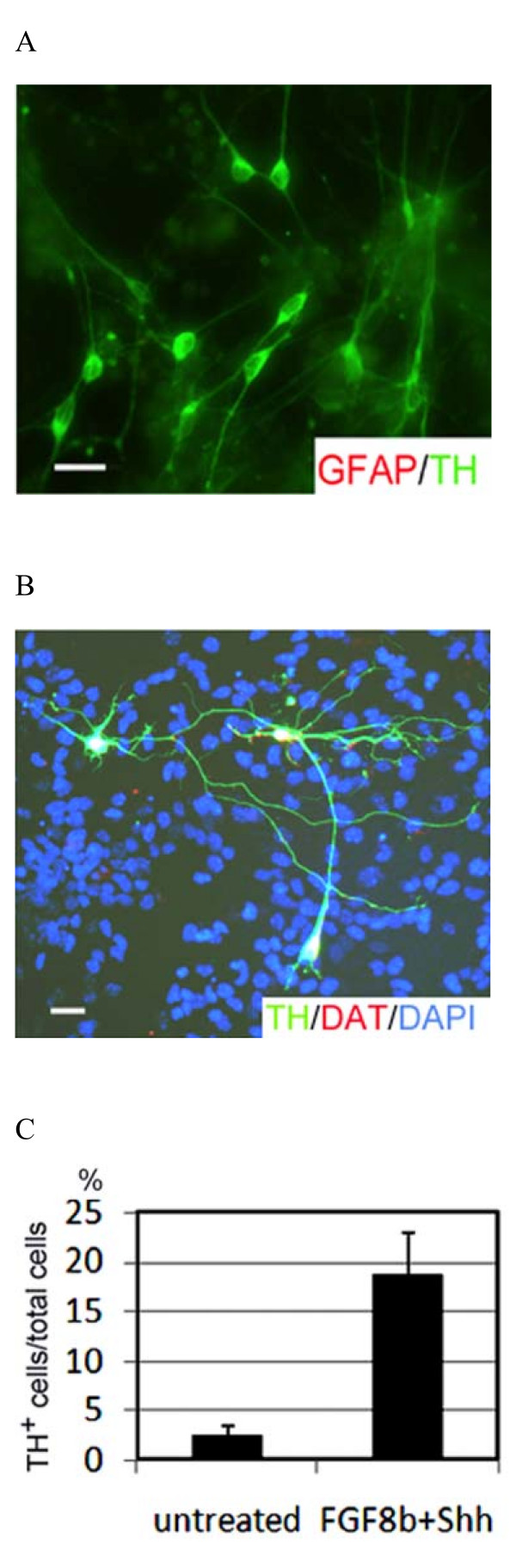
**The percentage of ES cells differentiates into TH-positive cells in vitro culture**. (A) GFAP (red) and tyrosine hydroxylase (green) immunostaining after exposure FGF8b and Shh. At this stage almost no GFAP-positive glia cells were detected in the differentiating ES cells on day 10. (B) TH and DAT staining are two markers of dopamiergic neurons. (C) The efficiencies of the dopaminergic neuron induction: 18.7 ± 4.3% (Shh/FGF8b-treated ES cells) v.s. 2.4 ± 1.1% (mock-treated ES cells). The TH-positive cells were manually counted in total cells. The scale bar is 15 μm.

On differentiation day 10, we counted the number of TH-positive neurons and total number of GFAP-immunopositive cells in the culture. At this stage almost no GFAP-positive glia cells were detected in ES cells (Figure 2A). The TH-positive cells were manually counted in total cells. We showed the efficiencies of dopaminergic neuron induction under SFEB condition, which were 18.7 ± 4.3% and 2.4 ± 1.1% in Shh/FGF8b-treated ES cells and mock-treated ES cells, respectively (Figure 2C).

### Histological evaluation of stem cell transplantation

At four weeks after 6-OHDA-lesioning and at eight weeks after transplantation, animals were perfusion-fixed transcardially with a fixative (4% paraformaldehyde in phosphate buffer). Brains were removed and cryosections were made. TH-positive cells in the substantia nigra and striatum were evaluated histologically.

**Figure 3:**
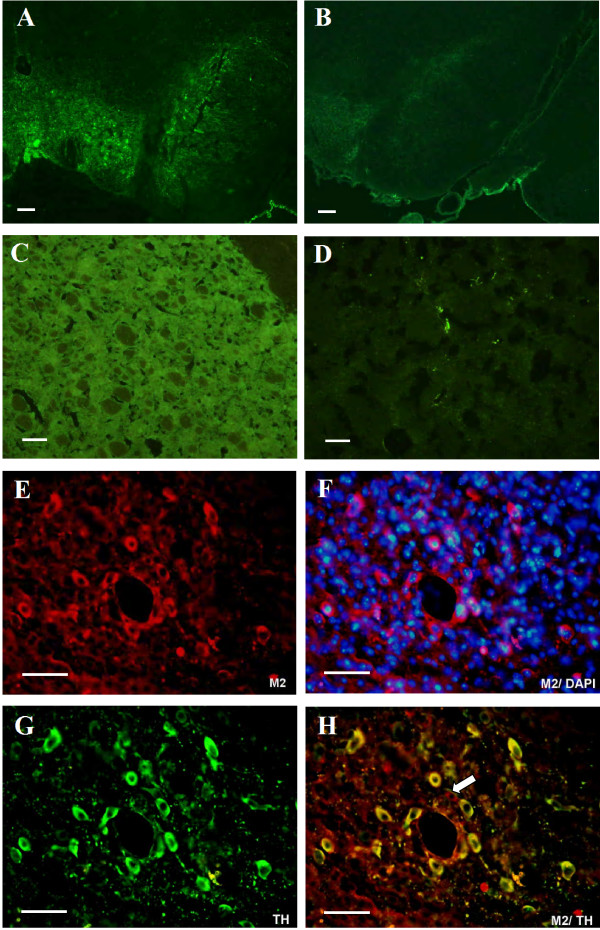
**The number of TH-positive cells was evaluated at four weeks after 6-OHDA lesioning**. Normal TH immunoreactivity was detected in the substantia nigra (A) and striatum (C) of all the sham animals. Depletion of TH immunoreactivity was noted in the substantia nigra (B) and striatum (D) at the side treated with 6-OHDA. Immunohistochemical staining was done at 8 weeks after implantation of ES-derived dopaminergic neurons into 6-OHDA lesioned striatum. The M2 mouse-specific antibody (E and F, red) and the nuclear marker DAPI (F, blue) were detected in numerous cells at the implantation site, suggesting that the grafts survived. TH-positive neurons were found within the graft (G, green). All TH-positive profiles coexpressed the M2 mouse-specific antibody (H, yellow), and one cell revealed M2 staining only (arrowhead). The scale bar: A, B: 200 μm; C to H: 50 μm.

Normal TH immunoreactivity was detected in substantia nigra (Figure 3A) and in the striatum (Figure 3C) of all sham animals. Depletion of TH immunoreactivity was noted in the substantia nigra (Figure 3B) and striatum (Figure 3D) at the side treated with 6-OHDA. We grafted ES-derived dopaminergic neurons into the rat striatum on the right side and then examined graft markers at eight weeks using immunofluorescence. The M2 mouse-specific antibody (Figure 3E, red) and the nuclear marker DAPI (Figure 3F) were detected in numerous cells at the implantation site, suggesting that the grafts survived. At 8 weeks post-transplantation, engrafted dopaminergic neurons expressed tyrosine hydroxylase (Figure 3G) and co-labeled by the M2 antibody (Figure 3H, yellow coexpression).

## Discussion

The present study demonstrated that the CatWalk method is useful for analyzing gait changes in rats after unilateral 6-OHDA lesioning and dopaminergic neuron grafting. Some of the dynamic and static parameters were altered in rats with 6-OHDA-induced lesions including paw contact pressure and area, velocity, and hindlimb support. There was no significant change in inter-limb coordination. Furthermore, engrafting dopaminergic neurons into the striatum on the lesion side partially improved gait impairments.

In a distal middle cerebral artery occlusion model of focal ischemic stroke, Wang Y. et al. found a persistent reduction in paw pressure and maximal area of paw contact. They attributed these findings to an altered use of the plantar surface [13]. Similar results were noted in a model of unilateral spinal cord injury, in which the maximal contact area of the affected fore paws was significantly reduced because of reduced forelimb weight bearing [20]. Using the CatWalk-automated gait analysis method, we found that unilateral dopamine deficiency led to a persistent reduction in paw pressure and print area of paw contact in the affected limbs. This was most likely due to the rigidity of muscle tone and altered use of paw surface. The intensity and contact area of the unaffected paws of 6-OHDA-lesioned rats tended to decrease as well, possibly because they were compensating for the affected limbs. These findings have also been reported in a focal ischemic stroke model [13].

Swing duration and swing speed are related to velocity during locomotion analysis [21]. Wang Y. et al. reported that temporal parameters such as stance, swing, and stride length remained largely unchanged in their middle cerebral artery occlusion model of focal ischemic stroke most likely because these parameters do not affect velocity in stroke [13]. An increase in swing duration and a decrease in swing speed of the hindlimbs has been reported in animals with bilateral 6-OHDA-induced lesions [22]. Similar results were noted in our unilateral dopaminergic neuron-deficient model. We observed that swing speed of all four paws was slower, and that the velocity of the hindpaws was faster than the swing speed and velocity of the forepaws during the swing phase. One possible explanation for this finding is that the rats were using their unaffected paws to compensate for the impaired gait caused by affected limbs.

Unstable gait can be compensated by a short swing duration and large base-of-support (BOS) [11]. The base-of-support of the hind limbs was shown to increase by up to 50% following bilateral hemisection of dorsal spinal cord in rats [23]; however, in an inflammatory pain model, it was found that carrageenan-induced right knee inflammation did not significantly change the BOS of the hind paws [14]. Our results, however, demonstrated that the distance between of the two hind paws increased after injection of 6-OHDA. We hypothesize that the distance between the rear paws increased in order to compensate for the affected limbs.

Inter-limb coordination is a tool to study neural control of locomotion [24]. The phase dispersion, step pattern, and regularity index are common parameters for measuring the degree of inter-limb coordination. Phase dispersion describes the temporal relationship between placements of two paws within a step cycle [25]. The phase dispersion describes the relationship between the initial contact of one paw (target paw) to the stride cycle of another paw (anchor paw) and is expressed as a percentage. A phase dispersion that exceeds 75% indicates that the target paw is more closely associated in time with the next anchor paw [26]. In an ischemic stroke model, Wang Y. et al. reported that the phase dispersion of the girdle pair between LH_anchor _and RH_target _paws was greater in experimental animals than in control animals [13]. In a sciatic nerve resection model, the affected hind paw was placed later relative to contralateral paw [25], and similar results were noted in an inflammatory pain model following the administration of carrageenan [14]. Our results demonstrated no differences in phase dispersion of the girdle pairs, ipsilateral pairs, or diagonal pairs before and after 6-OHDA lesioning.

Step pattern is another parameter related to interlimb coordination. The 'Ab' alternate pattern is the most common step cycle in intact rats [27]. The Ab pattern remains predominant in animals subjected to spinal cord injury or cerebral artery occlusion [11,13]. In a rat model of left sciatic nerve resection, however, it was found that the alternate 'Aa' and cruciate 'Ca' patterns decreased, but that the 'Ab' and 'Cb' paterns increased in rats after the resection [25]. In our study, there was no significant change in the Ab pattern after the unilateral infusion of 6-OHDA. The regularity index is another relevant parameter reflecting inter-limb coordination. In a spinal transaction injury model, RI temporarily decreased during the early post-surgical stage but partially recovered in the late post-surgical stage [10]. In our study, there was no change in RI after 6-OHDA lesioning. PD patients adapt their coordination patterns by manipulating walking speed in the early stage. However, this adaption is limited because of rigidity and bradykinesia and is related to the degeneration of the dopaminergic system. Less adaption between movement of arms and legs was observed in PD patients as compared with healthy controls [28]. In the present study, unilateral 6-OHDA infusions did not alter the inter-limb coordination parameters. This is likely because the adaptation of limbs and trunk during moving differ between quadrupeds and humans with PD.

The obvious symptom observed in human PD is commonly associated with an average loss of dopaminergic neurons in the substantia nigra in the range of 60-80%, and reduction of dopamine level by over 95% in striatum [29]. In modelling PD for preclinical research, injection of the neurotoxin 6-OHDA into medial forebrain bundle results in near total depletion of dopamine in the ipsilateral sriatum [30]. After 6-OHDA injection into the medial forbrain bundle, dopaminergic neurons began to die within the first day. Studies have shown that within 3-4 days after lesioning, reduction of dopamine level in striatum reached the maximum and the residual striatal dopamine level was less than 20% of the control level [31,32]. An imbalance in dopamine activity between the bilateral striatum causes rotation asymmetry after unilateral 6-OHDA lesioning, which causes animals to rotate away from the side of greater activity. Thus, administration of the dopamine-releasing agent methamphetamine produces ipsilateral rotations (rotation toward the 6-OHDA lesion side) because it induces an increased release of dopamine into the non-lesioned nigrostriatal projection. The magnitude of asymmetric circling motor behaviour depends on the degree of nigrostriatal lesioning [33]. Quantification of this circling behaviour has been used extensively to evaluate the anti-parkinsonian potential of new drugs, gene theapies, and transplantation [34,35]. In this study, we showed that transplantation of dopaminergic neurons derived from embryonic stem cells into 6-OHDA-lesioned rodents improved drug-induced rotational scores over time. We also observed that the paw contact area, paw pressure, and swing speed improved, and that the BOS of the hind limbs decreased after dopaminergic neuron grafting. Otherwise, unilateral 6-OHDA infusions led to gait disturbance not only in the affected side paws but also in the unaffected paws. We hypothesize that the rats changed the use of the unaffected paw to compensate for the affected paws in order to maintain a straight path down the narrow glass walkway.

A previous report suggested that body weight exceeding 300 to 350 g might affect the value of paw print length and toe spread [36]. Koopmans et al found differences of gait parameters among different strains of rats of the same age [37]. In an ischemic stroke model, there is no significant correlation between body weight and any gait parameter, including intensity and contact area [13]. To determine whether body weight affected the measures of gait, we conducted Pearson's correlation analysis at four weeks after 6-OHDA lesioning. Body weight did not affect paw pressure or contact area in sham or 6-OHDA-lesioned rats (data not shown), possibly because of a small variation in body weight and a lack of difference in strain.

Various strategies have been employed to try to increase the yield of dopamine neurons from cultured ES cells. Kim et al. reported that a midbrain dopaminergic phenotype with TH-positive neurons was promoted by expressing nuclear receptor related-1 (Nurr1) with adding the trophic factors FGF8 and SHH [38]. We transplanted FGF8b- and SHH-treated Sox1-GFP knock-in embryonic stem cells into the striatum on the lesion side. Four weeks after transplantation, we found numerous M2-positive cells in the striatum, indicating that the grafts survived. A few TH-positive immunoreactive cells were noted on the lesion side at eight weeks after transplantation.

As PD advances, gait disorders become more pronounced, leading to the potential for falls and the associated sequelae. A prospective study found that 50.8% of people with PD experienced at least one fall in a six-month period and 25.4% of patients experienced recurrent falls [39]. These falls might result in hip fracture, vertebral compression fracture and head injury, increasing the risk of bedridden condition and mortality rate. Systematic reviews of longitudinal studies have revealed that posture instability and gait difficulty predict future disability in PD [40]. Thus, preventing disability and improving quality of life by ameliorating gait disturbance are important for therapy.

The GaitRite system, which is very similar to the CatWalk system, has been used to detect footfall patterns, as well as selected time and distance measurements of persons with early-stage Parkinson's disease [41]. The GaitRite system comprises an electronic walkway embedded with many pressure sensors from which to collect data on spatial and temporal gait parameters while crossing the walkway. Using this system, Nelson et al. found that cadence was reduced in patients with PD. Similar results were observed in our animal model of PD. Paquet et al. used an accelerometric device designed for human locomotion analysis to compare the gait of patients with Parkinson's disease with that of healthy individuals, and found that walking velocity, stride frequency, step length, and walking regularity were markedly reduced in patients with PD [42]. The Pedar insole system, which comprises 99 capacitive transducers on flexible insoles, was used by Kimmeskamp and Hennig to determine the in-shoe pressure distribution during normal gait in patients with PD and in healthy individuals. The researchers found that patients with PD show significant changes in foot loading behaviour with reduced peak pressures in the lateral heel region [43]. The features of Parkinsonian gait are probably manifestations of adaptive mechanisms to avoid unsteadiness and falling during walking.

In conclusion, the CatWalk-assisted automated gait analysis system revealed that unilateral infusion of 6-OHDA leads to functional changes in static and dynamic gait parameters. Furthermore, the CatWalk system showed that grafting of ES-derived dopaminergic neurons into the striatum partially reduces gait impairments, at least in part, by dopaminergic replacement.

## Abbreviations

PD: Parkinson's disease; 6-OHDA: 6-hydroxydopamine; TH: tyrosine hydroxylase; ES cells: embryonic stem cells; FGF8b: Fibroblast Growth Factor 8b; SHH: Sonic hedgehog homolog.

## Competing interests

The authors declare that they have no competing interests.

## Authors' contributions

CSC carried out the main experiment and drafted the manuscript; HLS designed the experiment and helped to drafted the manuscript; FCC helped to gait analysis and Immunohistochemical assay; ShH helped to create animal model and culture of stem cells; CFC helped to finish animal model and data collection; and CSL are responsible for study design and statistic analysis. All authors read and approved the final manuscript.
